# Evaluation of the effects of microencapsulated DL-methionine on productive performance, immunity, plasma amino acids, and hepatic growth-related gene expression in broilers

**DOI:** 10.1016/j.vas.2026.100593

**Published:** 2026-02-06

**Authors:** Mohammad Ali Khazab, Hossein Ali Ghasemi, Seyed Abdullah Hosseini, Iman Hajkhodadadi, Amir Hossein Alizadeh-Ghamsari

**Affiliations:** a Department of Animal Science, Faculty of Agriculture and Environment, Arak University, Arak 38156-8-8349, Iran; b Department of Animal and Poultry Nutrition and Physiology, Animal Science Research Institute of Iran, Agricultural Research, Education and Extension Organization (AREEO), Karaj, Iran

**Keywords:** DL-methionine, Microencapsulation, Broiler performance, Antibody response, Amino acid profile, Hepatic growth genes

## Abstract

A 42-day feeding trial was conducted to compare a diet supplemented with crystalline DL-methionine (**control**) with diets containing microencapsulated DL-methionine (**MEM**) supplied at 60, 70, 80, 90, or 100 % of the commercially recommended level in broiler chickens. A total of 1260 one-day-old Arian broilers were randomly allotted to six treatments, with seven replicates of 30 birds each. Body weight gain (**BWG**), feed intake, and feed conversion ratio (**FCR**) did not differ between birds fed the control diet and those fed MEM diets containing ≥70 % of the recommended level, whereas the 60 % MEM diet reduced BWG and increased FCR. Increasing the dietary MEM level resulted in a quadratic increase in leg yield and a linear decrease in abdominal fat, without affecting hematological traits or leukocyte differential counts. The 100 % MEM diet increased influenza (H9N2) antibody titers, and all MEM levels enhanced Newcastle disease virus titers compared with the 60 % MEM diet; moreover, titers in the 100 % MEM group also exceeded those of the control. Plasma methionine, serine, and total amino acid concentrations were higher in birds fed the 60 % MEM diet than in those fed the 90 % MEM diet, while plasma cysteine showed a quadratic response as MEM level increased. Diets providing 80–100 % MEM upregulated hepatic GHR expression, and diets providing 90–100 % MEM also increased mTOR expression compared with the 60 % MEM diet. Overall, microencapsulated DL-methionine enabled approximately a 30 % reduction in supplemental methionine without impairing performance, while higher inclusion levels (≥80 % of the recommended level) beneficially modulated immune responses and growth-related gene expression.

## 1 Introduction

Broiler chickens require a balanced diet that provides essential nutrients for growth, with amino acids (**AA**) being particularly critical for protein synthesis, muscle development, and overall growth (Selle et al., 2023; Xie et al., 2024). Methionine (**Met**), a key amino acid, is the first-limiting amino acid in corn–soybean meal-based diets (Izadi et al., 2024). Beyond its role in protein synthesis, Met also regulates several metabolic pathways and influences growth signaling (Gong et al., 2025). The somatotropic axis, comprising growth hormone (**GH**), its receptor (**GHR**), and insulin-like growth factor 1 (**IGF-1**), is central to growth regulation in poultry (Jia et al., 2018; Vaccaro et al., 2022). This axis promotes protein synthesis and cell growth via mechanistic target of rapamycin (**mTOR**) activation while inhibiting negative regulators such as myostatin (Wen et al., 2017a; Xu & Velleman, 2023). Ndunguru et al. (2024) reported that a mild increase in embryonic Met activates IIS/mTOR signaling, increases hepatic IGF-1 and mTOR expression, elevates circulating IGF-1 later in development, and leads to faster post-hatch growth, thereby linking higher Met levels to enhanced mTOR phosphorylation and muscle growth. Additionally, Met deficiency reduces somatotropic axis gene expression, including GHR and IGF-I, shifting the balance toward protein degradation (Zeitz et al., 2019; Klünemann et al., 2024). Furthermore, previous studies have shown that both the source and level of Met influence growth and amino acid metabolism, with higher Met levels improving growth and intestinal health (Maynard et al., 2023; Liu et al., 2024).

Encapsulating poultry feed supplements, particularly AA such as Met, has become a popular strategy to enhance the efficiency and bioavailability of these nutrients (Sun et al., 2020a, b). Encapsulation helps protect bioactive compounds, such as AA, from environmental degradation, including oxidation, and ensures a controlled release in the digestive system, improving nutrient absorption and reducing wastage (Zabot et al., 2022; Seleem et al., 2024). Traditional encapsulation techniques have provided benefits in terms of amino acid stability, but recent advances in microencapsulation have offered superior advantages. Microencapsulation involves enclosing AA within a protective coating, often made of lipids or other biodegradable polymers, that resists degradation under challenging conditions, like low pH in the stomach, but releases the active ingredient slowly in the intestine (Collins, 2021; Contreras-López et al., 2024). This slow-release mechanism can improve the utilization of AA, minimize losses due to leaching, and enhance metabolic benefits, such as better regulation of amino acid absorption and metabolism (Liu et al., 2024). Other animal studies have shown that microencapsulated Met and other AA are more efficiently utilized compared with traditional crystalline forms, leading to improved performance and amino acid utilization (Guo et al., 2020; King et al., 2021). However, available broiler studies on microencapsulated DL-Met have often focused on single inclusion levels and provide limited evidence on dose–response replacement strategies combined with mechanistic outcomes related to methionine utilization (e.g., plasma free amino acid profiles and hepatic growth-related signaling). In addition, microencapsulation matrices differ in release kinetics and may alter effective bioavailability. Therefore, the present study evaluated a newly developed microencapsulated DL-Met product across graded inclusion rates.

Given the advantages of controlled release, we hypothesized that a newly developed microencapsulated DL-Met product could allow a partial reduction in supplemental methionine relative to crystalline DL-Met while maintaining productive performance and beneficially modulating immune responses, plasma amino acid balance, and hepatic growth-related gene expression. It is also expected that broilers supplemented with microencapsulated Met will show improved growth performance and carcass traits compared with those fed crystalline DL-Met, with the enhanced performance likely due to the slow-release properties of microencapsulation, leading to better Met absorption, amino acid balance, protein synthesis regulation, and activation of growth-related pathways like mTOR and IGF-I secretion. Accordingly, the objective of this study was to investigate, in a dose–response replacement design, the effects of graded supplementation of a newly developed microencapsulated DL-Met product (60–100 % of the commercial recommendation) compared with crystalline DL-Met (100 %) on growth performance, carcass traits, hematological indices, antibody response, plasma free amino acid profile, and hepatic expression of growth-related genes (mTOR, GHR, IGF-1) in broilers.

## 2 Materials and methods

### 2.1 Animal ethics

The experimental protocol adhered to the guidelines set by the Animal Ethics Committee of Arak University (Approval number 1402-d-13,327) and followed the ARRIVE guidelines 2.0 for animal research, ensuring ethical practices in the treatment and care of the animals. Prior to the initiation of the study, all relevant ethical considerations were thoroughly reviewed, and appropriate steps were taken to minimize animal suffering.

### 2.2 Birds and treatments

A total of 1260 one-day-old Arian broiler chicks were individually weighed at placement (day 0) and then allocated to 42 floor pens (30 birds per pen) in a manner that balanced the mean initial body weight (BW) across pens (i.e., chicks were distributed based on initial weighing to achieve BW uniformity among pens). After pen formation, the 42 pens were randomly assigned to the six dietary treatments (7 pens per treatment) using a random allocation procedure. The six treatments included varying levels of microencapsulated Met supplementation (60 %, 70 %, 80 %, 90 %, and 100 % of the commercial recommendation), compared with a control treatment with crystalline DL-Met at 100 % of the commercial level. The experimental units consisted of 42 pens, each measuring 4.5 m² (2.5 × 1.8 m). The broilers were housed under controlled environmental conditions, with a lighting regimen of 23 h of light and 1 h of darkness per day. The temperature was maintained at 33 °C for the first 48 h prior to chick arrival, after which it was gradually reduced by approximately 2.5 °C per week to simulate natural environmental changes as the birds aged. Relative humidity was maintained at 60 % throughout the study.

The Met used in this study was provided as a high-purity crystalline product (Evonik, Essen, Germany) with the chemical formula C₅H₁₁NO₂S and a molecular weight of 149.21 g/mol, in crystalline powder form with no odor, and a purity of over 98 % (as per the certificate of analysis and confirmed by chromatographic analysis, high-performance liquid chromatography [HPLC]). The microencapsulated DL-Met (MEM) used in this study was manufactured by a knowledge-based company (Soroush Sabz Alborz, Karaj, Iran) and was used as received. According to the manufacturer, MEM consists of DL-Met particles coated with a proprietary protective matrix designed to improve handling stability and modulate release; therefore, the exact coating composition and processing parameters (embedding material identity and preparation conditions) are confidential to the manufacturer. For reproducibility, the following product specifications were provided: MEM maintained stability at temperatures up to 80 °C, with a mean particle size of 25 ± 6 µm after encapsulation. The active DL-Met content was 72 %, as confirmed by HPLC analysis. The product was stored in sealed containers under dry conditions at ambient temperature until use. Experimental diets were formulated to provide Met levels ranging from 60 % to 100 % of the commercially recommended levels. The feed ingredients and chemical composition of the experimental diets for the different rearing phases are presented in Table 1, Table 2.

**Table 1:** Ingredients and nutrient composition of the experimental diets (days 1–21).

	Dietary Treatments^1^					
Ingredient (%, as‑fed)	Control	MEM60	MEM70	MEM80	MEM90	MEM100
Corn	56.53	56.53	56.53	56.53	56.53	56.53
Soybean meal	34.92	34.92	34.92	34.92	34.92	34.92
Corn gluten meal	1.78	1.78	1.78	1.78	1.78	1.78
Soybean oil	1.50	1.50	1.50	1.50	1.50	1.50
Dicalcium phosphate	2.18	2.18	2.18	2.18	2.18	2.18
Calcium carbonate	1.22	1.22	1.22	1.22	1.22	1.22
Sodium chloride	0.23	0.23	0.23	0.23	0.23	0.23
Sodium bicarbonate	0.08	0.08	0.08	0.08	0.08	0.08
DL-Met (98%)	0.31	—	—	—	—	—
Microencapsulated DL-Met (72%)	—	0.25	0.30	0.34	0.39	0.43
L-lysine HCl	0.33	0.33	0.33	0.33	0.33	0.33
L-threonine	0.23	0.23	0.23	0.23	0.23	0.23
Mineral premix	0.25	0.25	0.25	0.25	0.25	0.25
Vitamin premix	0.25	0.25	0.25	0.25	0.25	0.25
Inert filler (silica sand)	0.19	0.25	0.20	0.16	0.11	0.07
Calculated nutrient composition						
Metabolizable energy (kcal/kg)	2902	2902	2902	2902	2902	2902
Crude protein (%)	22.1	22.1	22.1	22.1	22.1	22.1
Calcium (%)	1.04	1.04	1.04	1.04	1.04	1.04
Available phosphorus (%)	0.52	0.52	0.52	0.52	0.52	0.52
Sodium (%)	0.16	0.16	0.16	0.16	0.16	0.16
Digestible Met (%)	0.62	0.49	0.53	0.56	0.59	0.62
Digestible Met + Cys (%)	0.93	0.80	0.84	0.87	0.90	0.93
Digestible lysine (%)	1.22	1.22	1.22	1.22	1.22	1.22
Digestible threonine (%)	0.85	0.85	0.85	0.85	0.85	0.85
Dietary electrolyte balance (mEq/kg)	250	250	250	250	250	250
Analyzed value,%						
Crude protein	21.5	21.5	21.6	21.6	21.5	21.6
Total Met	0.66	0.52	0.55	0.59	0.62	0.63
Total Met + Cys	1.00	0.87	0.91	0.94	0.97	1.01

1 Microencapsulated DL‑Met (MEM) treatments; MEM60–MEM100 were formulated with microencapsulated DL‑Met (72 %) at 60 %, 70 %, 80 %, 90 %, and 100 % of the commercially recommended Met level, respectively. The Control diet contained DL‑Met (98 %) at the commercially recommended level. Values are on an as‑fed basis unless otherwise indicated.

**Table 2:** Ingredients and nutrient composition of the experimental diets (days 22–42).

	Dietary Treatments^1^					
Ingredients (%, as‑fed)	Control	MEM60	MEM70	MEM80	MEM90	MEM100
Corn	64.82	64.82	64.82	64.82	64.82	64.82
Soybean meal	28.72	28.72	28.72	28.72	28.72	28.72
Corn gluten meal	—	—	—	—	—	—
Soybean oil	2.03	2.03	2.03	2.03	2.03	2.03
Dicalcium phosphate	1.65	1.65	1.65	1.65	1.65	1.65
Calcium carbonate	1.06	1.06	1.06	1.06	1.06	1.06
Sodium chloride	0.20	0.20	0.20	0.20	0.20	0.20
Sodium bicarbonate	0.13	0.13	0.13	0.13	0.13	0.13
DL-Met (98 %)	0.25	—	—	—	—	—
Microencapsulated DL-Met (72 %)	—	0.21	0.24	0.28	0.31	0.34
L-lysine HCl	0.28	0.28	0.28	0.28	0.28	0.28
L-threonine	0.21	0.21	0.21	0.21	0.21	0.21
Mineral premix	0.25	0.25	0.25	0.25	0.25	0.25
Vitamin premix	0.25	0.25	0.25	0.25	0.25	0.25
Inert filler (silica sand)	0.16	0.20	0.17	0.13	0.10	0.07
Calculated nutrient composition						
Metabolizable energy (kcal/kg)	3017	3017	3017	3017	3017	3017
Crude protein (%)	18.9	18.9	18.9	18.9	18.9	18.9
Calcium (%)	0.85	0.85	0.85	0.85	0.85	0.85
Available phosphorus (%)	0.42	0.42	0.42	0.42	0.42	0.42
Sodium (%)	0.16	0.16	0.16	0.16	0.16	0.16
Digestible Met (%)	0.51	0.42	0.44	0.47	0.49	0.51
Digestible Met + Cys (%)	0.78	0.69	0.71	0.74	0.76	0.78
Digestible lysine (%)	1.04	1.04	1.04	1.04	1.04	1.04
Digestible threonine (%)	0.74	0.74	0.74	0.74	0.74	0.74
Dietary electrolyte balance (mEq/kg)	230	230	230	230	230	230
Analyzed value,%						
Crude protein	18.4	18.5	18.5	18.6	18.5	18.4
Total Met	0.55	0.45	0.47	0.49	0.52	0.54
Total Met + Cys	0.85	0.76	0.78	0.80	0.83	0.86

1 Microencapsulated DL‑Met (MEM) treatments; MEM60–MEM100 were formulated with microencapsulated DL‑Met (72 %) at 60 %, 70 %, 80 %, 90 %, and 100 % of the commercially recommended Met level, respectively. The Control diet contained DL‑Met (98 %) at the commercially recommended level. Values are on an as‑fed basis unless otherwise indicated.

### 2.3 Growth performance and slaughter traits

Performance parameters, including body weight gain (**BWG**), feed intake (**FI**), and feed conversion ratio (**FCR**), were recorded weekly. BWG and FI were measured at the beginning and end of each week. FCR was calculated for each week and for the total experimental period. To correct for the effects of mortality, the performance data were adjusted for the mortality rate, where the FI and BWG were corrected based on the number of birds surviving at each time point.

At the end of the 42-day experimental period, two birds per replicate pen were randomly selected for slaughter and dissection. The birds were euthanized humanely by cervical dislocation, and the carcass and its components were weighed. Slaughter traits, including carcass yield (as a percentage of live weight), breast, leg, back, heart, liver, spleen, bursa, gizzard, and abdominal fat, were measured. The data were used to calculate the percentage of each organ and muscle relative to live body weight.

### 2.4 Hematological characters

At 42 days of age, blood samples were collected from two birds per replicate pen into EDTA-anticoagulated tubes. Red blood cell (**RBC**) and white blood cell (**WBC**) counts were determined using a hemocytometer with Natt-Herrick solution as a diluent stain. Hemoglobin concentration was measured by the cyanmethemoglobin method, and hematocrit (Hct) was determined using the microhematocrit method. To assess the leukocyte profile, 100 leukocytes were counted under an optical microscope (BX51; Olympus, Tokyo) to determine the percentage of heterophils and lymphocytes. The heterophil-to-lymphocyte ratio (**H/L**) was calculated as described by Lucas and Jamroz (1961). These measurements were used to assess the overall health status and immune function of the birds, following the protocol described by Omidi et al. (2015).

### 2.5 Plasma free amino acids analysis

At the end of the experimental period, blood samples were collected from two birds per replicate (selected as those closest to the median pen body weight) using heparinized tubes to prevent clotting. The plasma was separated by centrifugation at 2500 × *g* for 15 min at 4 °C, and samples were stored at −80 °C until analysis. Plasma samples were deproteinized by combining equal volumes of plasma and a 7.5 % (w/v) trichloroacetic acid solution in a 1.5-ml microcentrifuge tube. The mixture was vortexed for 30 s and then centrifuged at 15,000 × *g* for 15 min. The resulting supernatant was transferred into screw-cap cryovials and stored at 4 °C. The supernatant was then analyzed for amino acid quantification using an automatic amino acid analyzer (Hitachi l-8800, Tokyo, Japan), which was equipped with a ninhydrin reagent, a lithium buffer system, and photometric detection of individual AA. Plasma uric acid was analyzed using an automatic biochemical analyzer (Clima; Ral. Co, Barcelona, Spain) using a commercial kit (Pars Azmoon Co., Tehran, Iran).

### 2.6 Antibody response

To measure total antibody titers against Newcastle Disease Virus (**NDV**) and avian influenza virus (**AIV**) vaccines, broiler chickens were vaccinated with the live Newcastle B1 strain (Avishield® ND B1, Genera Inc., Croatia) on day 7 and injected subcutaneously into the dorsal region of the neck with a killed Newcastle + Influenza (H9N2) commercial vaccine (Gallimune 208 ND+ Flu H9 ME, Merial Inc., France). A single blood sample was collected on day 21 by wing vein puncture to obtain serum. The hemagglutination inhibition (HI) test was performed to determine antibody titers. Serum samples were serially diluted in saline, starting from a 1:2 dilution, and mixed with an equal volume of the respective virus antigen. After incubating for 30 min at room temperature, a RBC suspension was added to each well, and the mixture was incubated for an additional 30 min. The highest dilution of serum that inhibited hemagglutination was recorded as the antibody titer, with the results expressed as the geometric mean of the titers (Talazadeh et al., 2022).

### 2.7 Gene expression measurement

To evaluate the expression of growth-related genes, liver tissue samples were collected (*n* = 7 pert treatment). Following euthanasia, a 2–3 cm section from the mid-region of the liver was excised, washed with cold phosphate-buffered saline, immediately frozen in liquid nitrogen, and stored at −80 °C until RNA extraction. Total RNA was extracted using an RNA extraction kit (Pars-Tous, Mashhad, Iran) according to the manufacturer’s instructions. The purity and quantity of RNA were assessed using a spectrophotometer (A260/A280), and samples were stored at −80 °C until cDNA synthesis. To eliminate genomic DNA contamination, RNA was treated with DNase I, and cDNA synthesis was performed using a cDNA synthesis kit (SinaClon, Tehran, Iran). A total of 25 ng of cDNA template was used in each reaction, which was carried out in the presence of SYBR Green qPCR Master Mix (containing ROX). Thermal cycling conditions were set according to the MIQE guidelines and standard procedures. The target genes included mechanistic target of rapamycin (**mTOR**), growth hormone receptor (**GHR**), insulin like growth factor 1 (**IGF-1**), and the reference gene glyceraldehyde-3-phosphate dehydrogenase (**GAPDH**). Specific primers for each gene, including amplicon length and GenBank accession numbers, are listed in Table 3. Relative gene expression was quantified using the 2^-ΔΔCt^ method (Livak & Schmittgen, 2001), where the Ct value of the target gene was normalized to the Ct value of GAPDH. The ΔΔCt value was calculated relative to the control group, ensuring that gene expression was accurately compared across treatments.

**Table 3:** List of primers used for real-time PCR.

Gene^1^	Primer sequence 5′−3′	Length (nt)	GenBank number
mTOR	F: TTGGGTTTGCTTTCTGTGGCTGTC	119	XM_417,614
	R: ACAGACTTCTGCCTCTTGTGAGCA		
GHR	F: GCTGCATCATATCAGGGTTCTTC	161	XM_046934916.1
	R: GTCCTGTAGCCCTTGGTCAC		
IGF-1	F: CTGGTTGATGCTCTTCAGTTCG	142	NM_001004384.3
	R: AGCCTCCTCAGGTCACAACTCT		
GAPDH	F: CAGAACATCATCCCAGCGTCCAC	134	NM_204,305.2
	R: CGGCAGGTCAGGTCAACAACAG		

1 **MTOR**, mechanistic target of rapamycin; **GHR**, growth hormone receptor; **IGF1**, insulin-like growth factor 1; **GAPDH**, glyceraldehyde-3-phosphate dehydrogenase (housekeeping gene).

### 2.8 Statistical analysis

Data were analyzed using a completely randomized design with 6 treatments and 7 replicates, with the experimental unit being the pen. Treatment effects were estimated using one-way analysis of variance (PROC GLM) in SAS software (version 9.4, SAS Institute, Cary, North Carolina). If treatment effects were significant (*P* < 0.05), means were compared using Tukey post hoc tests. Trend analysis was conducted only for the five levels of microencapsulated DL-Met (MEM60, MEM70, MEM80, MEM90, and MEM100). For this purpose, orthogonal polynomial contrasts (linear and quadratic) with corresponding coefficients for equal spacing were defined. The significance threshold was set at α = 0.05, with trends considered significant for 0.05 ≤ *P* < 0.10. In tables, mean ± SEM, P-values for treatment effects, and linear and quadratic statistics were reported for the MEM levels.

## 3 Results

### 3.1 Growth performance

The results related to growth performance from day 1 to day 42, categorized by week, are presented in Table 4. Up to day 7, the BWG, FI, and FCR were not significantly affected by the experimental treatments (*P* > 0.05). In week 2, a reduction in FI was observed in the MEM60 and MEM80 treatments compared with the control (*P* < 0.05). Additionally, during the same week, a significant improvement in FCR was observed in the MEM80 and MEM100 treatments compared with the control (*P* < 0.05; linear response). In weeks 3 and 4, the BWG was significantly higher in the MEM90 treatment compared with MEM60 (*P* < 0.05; linear response), and a reduction in FCR was observed in week 4 for the MEM90 treatment compared with MEM60 (*P* < 0.05; linear response). In week 5, the MEM80 and MEM100 treatments showed higher BWG compared with the MEM60 and MEM70 treatments (*P* < 0.05). All experimental treatments, except MEM70, led to a reduction in FCR compared to MEM60 during week 5 of the growing period (*P* < 0.05; both linear and quadratic responses). In week 6, the MEM80 and MEM90 treatments showed higher BWG compared with MEM60. Moreover, the MEM90 treatment exhibited a higher BWG than MEM70 at the same age (*P* < 0.05). A reduction in FCR was also observed in the MEM80 to MEM100 treatments compared with MEM60 (*P* < 0.05). Overall, during the entire experimental period (1 to 42 days), the highest BWG and the lowest FCR were observed in the MEM90 treatment, which showed a significant difference compared to the control, MEM60, and MEM70 treatments (*P* < 0.05). Additionally, the MEM80 and MEM100 treatments showed higher BWG and lower FCR compared with the MEM60 treatment (*P* < 0.05; both linear and quadratic responses).

**Table 4:** Growth performance observed in broiler chickens fed diets with varying substitution levels of commercial DL-Met with capsulated DL-Met.

	Experimental groups^1^								Contrast^3^	
Item^4^	Control	MEM60	MEM70	MEM80	MEM90	MEM100	SEM	*P*-value^2^	Linear	Quadratic
Week 1										
BWG, g/bird	125	129	125	121	125	126	3.8	0.810	0.472	0.205
FI, g/bird	152	152	151	151	146	145	3.6	0.507	0.075	0.742
FCR	1.22	1.18	1.21	1.25	1.17	1.16	0.025	0.110	0.223	0.045
Week 2										
BWG, g/bird	212	202	204	205	211	216	3.9	0.096	0.007	0.485
FI, g/bird	287^a^	264^b^	276^ab^	258^b^	273^ab^	275^ab^	4.7	0.002	0.268	0.451
FCR	1.36^a^	1.31^abc^	1.35^ab^	1.26^c^	1.29^abc^	1.27^bc^	0.019	0.003	0.031	0.969
Week 3										
BWG, g/bird	425^ab^	410^b^	430^ab^	437^ab^	440^a^	436^ab^	6.9	0.049	0.009	0.062
FI, g/bird	609	597	610	604	605	610	6.8	0.740	0.391	0.765
FCR	1.44	1.46	1.42	1.38	1.38	1.40	0.021	0.089	0.035	0.106
Week 4										
BWG, g/bird	422^ab^	404^b^	410^ab^	410^ab^	439^a^	435^ab^	7.8	0.012	0.001	0.733
FI, g/bird	700	682	685	690	695	696	6.7	0.366	0.075	0.917
FCR	1.66^ab^	1.69^a^	1.67^ab^	1.68^ab^	1.59^b^	1.60^ab^	0.026	0.026	0.003	0.644
Week 5										
BWG, g/bird	750^ab^	691^c^	720^bc^	777^a^	783^a^	766^a^	8.4	<0.001	<0.001	<0.001
FI, g/bird	1486	1458	1466	1484	1482	1484	22.3	0.917	0.316	0.685
FCR	1.98^bc^	2.11^a^	2.04^ab^	1.91^c^	1.89^c^	1.94^bc^	0.028	<0.001	<0.001	0.002
Week 6										
BWG, g/bird	560^abc^	523^c^	540^bc^	584^ab^	593^a^	572^abc^	11.6	0.001	<0.001	0.017
FI, g/bird	1225	1193	1210	1221	1223	1222	23.7	0.923	0.335	0.613
FCR	2.19^abc^	2.28^a^	2.24^ab^	2.09^c^	2.07^c^	2.14^bc^	0.033	<0.001	<0.001	0.006
Total 42 days										
BWG, g/bird	2,492^bc^	2,359^d^	2,431^cd^	2,533^ab^	2,591^a^	2,551^ab^	19.5	<0.001	<0.001	0.001
FI, g/bird	4461	4346	4399	4407	4422	4432	39.5	0.455	0.126	0.598
FCR	1.79^ab^	1.84^a^	1.81^a^	1.74^bc^	1.71^c^	1.74^bc^	0.013	<0.001	<0.001	0.004

Means within a row not sharing the salme superscript are different at *P* < 0.05. Values are means of 7 replicates (pens) per treatment.

1 Control: basal diet supplemented with Dl-Met supplement at the commercial recommended level for Met; MEM60, MEM70, MEM80, MEM90, and MEM100 treatments: basal diet supplemented with microencapsulated DL-Met at the levels of 60, 70, 80, 90, and 100 % of the commercial recommended levels for Met.

2 Means within a row with different superscripts (a–d) differ (Tukey’s test, *P* < 0.05). SEM indicates the pooled standard error of the mean. *P*-treatment is the overall one-way ANOVA *P*-value comparing all six treatments.

3 *P*-linear and *P*-quadratic are orthogonal polynomial contrasts testing linear and quadratic trends only across MEM60–MEM100 (control excluded), using orthogonal polynomial contrasts.

4 **BWG**, body weight gain, **FI**, feed intake; **FCR**, feed conversion ratio.

### 3.2 Slaughter traits

Based on the results pertaining to slaughter characteristics (Table 5), the yields of carcass, breast, and back, as well as the relative weights of the liver, spleen, bursa, gizzard, and abdominal fat, were not affected by the experimental treatments (*P* > 0.05). In contrast, the leg yield was significantly lower (*P* < 0.05) in the MEM60 treatment than in the MEM90 treatment. However, the leg yield in the other treatments was intermediate and did not show any significant differences (*P* > 0.05) relative to MEM60 or MEM90 treatments. The relative weight of the heart was higher (*P* < 0.05) in the MEM60 and MEM100 treatments compared with the MEM80 treatment. The abdominal fat percentage was lower in the MEM90 and MEM100 treatments (*P* < 0.05). Based on orthogonal analysis, either leg yield or relative heart weight exhibited a quadratic response, while abdominal fat storage showed a linear response to increasing levels of microencapsulated DL-Met in the diet.

**Table 5:** Slaughter traits observed in broiler chickens fed diets with varying substitution levels of commercial DL-Met with capsulated DL-Met.

	Experimental groups^1^								Contrast^3^	
Item^4^	Control	MEM60	MEM70	MEM80	MEM90	MEM100	SEM	*P*-value^2^	Linear	Quadratic
Carcass	72.51	70.75	72.50	72.86	73.37	71.32	1.095	0.555	0.573	0.093
Breast	21.74	20.76	22.07	21.14	21.65	21.48	0.802	0.463	0.320	0.493
Leg	22.32^ab^	21.09^b^	22.37^ab^	23.24^ab^	23.46^a^	21.67^ab^	0.467	0.017	0.161	0.002
Back	27.28	27.72	26.41	27.22	28.56	26.71	0.613	0.223	0.932	0.801
Heart	0.580^ab^	0.622^a^	0.524^ab^	0.505^b^	0.585^ab^	0.619^a^	0.0270	0.031	0.545	0.003
Liver	2.12	2.26	2.02	2.10	2.22	2.52	0.155	0.311	0.186	0.092
Spleen	0.080	0.126	0.099	0.092	0.103	0.106	0.0126	0.241	0.395	0.133
Bursa	0.071	0.105	0.088	0.079	0.087	0.121	0.0183	0.453	0.632	0.132
Gizzard	2.13	2.52	2.53	2.33	2.68	2.76	0.285	0.664	0.506	0.541
Abdominal fat	1.42^ab^	1.67^a^	1.35^ab^	1.27^ab^	1.21^b^	1.14^b^	0.107	0.036	0.004	0.249

Means within a row not sharing the same superscript are different at *P* < 0.05. Values are means of 7 replicates (pens) per treatment.

1 Control: basal diet supplemented with Dl-Met supplement at the commercial recommended level for Met; MEM60, MEM70, CM80, MEM90, and MEM100 treatments: basal diet supplemented with microencapsulated DL-Met at the levels of 60, 70, 80, 90, and 100 % of the commercial recommended levels for Met.

2 Means within a row with different superscripts (a–d) differ (Tukey’s test, *P* < 0.05). SEM indicates the pooled standard error of the mean. *P*-treatment is the overall one-way ANOVA *P*-value comparing all six treatments.

3 *P*-linear and *P*-quadratic are orthogonal polynomial contrasts testing linear and quadratic trends only across MEM60–MEM100 (control excluded), using orthogonal polynomial contrasts.

4 Based on the live preslaughter body weight.

### 3.3 Hematology

The results related to hematological parameters (Table 6) indicate that hematological parameters examined, including RBC and WBC counts, hemoglobin concentration, hematocrit percentage, heterophil percentage, lymphocyte percentage, and the H/L ratio, were not significantly affected by the dietary treatments (*P* > 0.05).

**Table 6:** Hematological parameters observed in broiler chickens fed diets with varying substitution levels of commercial DL-Met with capsulated DL-Met.

	Experimental groups^1^								Contrast^3^	
Item^4^	Control	MEM60	MEM70	MEM80	MEM90	MEM100	SEM	*P*-value^2^	Linear	Quadratic
WBC (× 10^3^/μl)	21.53	23.53	20.70	24.13	22.03	23.93	1.950	0.753	0.722	0.580
RBC (× 10^6^/μl)	2.85	2.67	2.63	2.60	2.43	2.53	0.113	0.225	0.127	0.700
Hemoglobin (g/dL)	11.48	11.43	10.57	12.10	11.44	11.50	0.626	0.692	0.574	0.870
Hematocrit (%)	35.00	33.00	32.83	30.00	31.33	31.92	1.877	0.552	0.580	0.471
Heterophil (%)	20.50	21.00	20.25	21.25	22.25	23.75	1.491	0.587	0.153	0.457
Lymphocyte (%)	72.50	71.75	73.50	72.00	72.75	70.75	1.591	0.876	0.624	0.432
H/L	0.283	0.293	0.275	0.299	0.306	0.338	0.0257	0.601	0.189	0.426

Means within a row not sharing the same superscript are different at *P* < 0.05. Values are means of 7 replicates (pens) per treatment.

1 Control: basal diet supplemented with Dl-Met supplement at the commercial recommended level for Met; MEM60, MEM70, MEM80, MEM90, and MEM100 treatments: basal diet supplemented with microencapsulated DL-Met at the levels of 60, 70, 80, 90, and 100 % of the commercial recommended levels for Met.

2 Means within a row with different superscripts (a–d) differ (Tukey’s test, *P* < 0.05). SEM indicates the pooled standard error of the mean. *P*-treatment is the overall one-way ANOVA *P*-value comparing all six treatments.

3 *P*-linear and *P*-quadratic are orthogonal polynomial contrasts testing linear and quadratic trends only across MEM60–MEM100 (control excluded), using orthogonal polynomial contrasts.

4 **RBC**, red blood cell; **WBC**, white blood cell; **H/L**, Heterophil to lymphocyte ratio.

### 3.4 Plasma amino acid profile

The plasma amino acid profile results are presented in Table 7. Significant increases (*P* < 0.05) were observed in the plasma concentrations of Met, Ser, and total AA (TAA) in birds fed the MEM60 diet compared with those fed the MEM90 diet. The MEM60 group showed a lower concentration of plasma Ile compared to the MEM100 group. The plasma concentrations of Leu were lower in the MEM60 and MEM90 treatments than in the control treatment. All experimental groups, with the exception of the MEM90 treatment, showed higher plasma Cys concentration compared with the MEM80 treatment. The plasma Lys concentration in the control and MEM90 treatments was lower than that in the MEM60 treatment. In accordance with orthogonal analysis, the plasma concentrations of Ile, Met, Cys, Ser, and total AA demonstrated a quadratic increase (*P* < 0.05) as the inclusion levels of micro-encapsulated DL-Met in the diet increased. Furthermore, the concentration of Lys exhibited both linear and quadratic increases in response to varying levels of dietary microencapsulated DL-Met. However, the experimental treatments did not significantly affect the plasma concentrations of other AA and uric acid.

**Table 7:** Plasma amino acid and uric acid (nmol/mL) concentrations observed in broiler chickens fed diets with varying substitution levels of commercial DL-Met with capsulated DL-Met.

	Experimental groups^1^								Contrast^3^	
Item^4^	Control	MEM60	MEM70	MEM80	MEM90	MEM100	SEM	*P*-value^2^	Linear	Quadratic
Arg	439.4	423.2	395.9	442.9	418.0	428.7	31.13	0.908	0.744	0.973
His	129.6	152.3	146.3	119.1	104.9	124.9	13.85	0.211	0.053	0.249
Val	254.3	258.3	250.3	204.8	201.0	236.1	20.03	0.210	0.141	0.093
Ile	142.0^ab^	138.7^ab^	142.4^ab^	137.5^ab^	104.8^b^	153.7^a^	9.03	0.025	0.751	0.042
Leu	300.8^a^	219.7^b^	269.3^ab^	265.0^ab^	215.9^b^	264.1^ab^	15.45	0.009	0.427	0.370
Lys	321.7^b^	452.9^a^	384.9^ab^	350.7^ab^	318.4^b^	373.2^ab^	24.59	0.012	0.017	0.025
Met	91.1^ab^	92.1^a^	85.3^ab^	78.0^ab^	72.9^b^	94.9^a^	4.26	0.011	0.635	0.003
Thr	461.8	504.0	456.7	558.1	453.3	468.5	49.60	0.647	0.656	0.681
Phe	165.1	160.8	157.7	155.1	121.1	152.8	9.77	0.061	0.056	0.220
Trp	59.2	57.2	58.1	63.8	57.0	63.8	3.89	0.662	0.373	0.970
Total IAA^1^	2365.1	2459.2	2346.8	2375.0	2067.2	2360.7	109.05	0.234	0.152	0.222
Glu	259.7	265.6	251.0	287.5	247.0	255.4	14.85	0.469	0.627	0.604
Gln	899.4	938.2	819.3	831.8	774.9	813.7	38.71	0.073	0.028	0.105
Asp	35.3	29.8	31.2	37.7	39.9	35.3	3.30	0.297	0.096	0.236
Asn	236.6	221.0	238.8	244.1	227.3	227.7	14.75	0.880	0.971	0.346
Ala	860.8	852.9	819.4	913.4	879.0	932.3	55.07	0.727	0.241	0.833
Cys	67.4^ab^	65.3^ab^	66.3^ab^	57.8^c^	62.6^bc^	70.2^a^	1.43	<0.001	0.229	<0.001
Gly	493.2	417.4	452.8	471.6	535.8	517.7	30.96	0.133	0.017	0.627
Ser	536.2^ab^	705.7^a^	624.6^ab^	572.9^ab^	505.6^b^	634.5^ab^	38.79	0.021	0.066	0.021
Tyr	241.1	280.0	220.7	260.1	245.5	215.4	29.78	0.658	0.258	0.968
Pro	389.2	479.9	405.6	402.6	395.5	438.4	21.83	0.069	0.234	0.020
Total DAA	4018.8	4255.8	3929.5	4079.6	3913.1	4140.4	93.09	0.132	0.421	0.041
Total AA	6383.9^ab^	6715.0^a^	6276.3^ab^	6454.5^ab^	5980.3^b^	6501.1^ab^	130.87	0.021	0.081	0.015
Uric acid	640.9	675.1	660.2	515.5	576.9	646.5	49.43	0.233	0.030	0.142

Means within a row not sharing the same superscript are different at *P* < 0.05. Values are means of 7 replicates (pens) per treatment.

1 Control: basal diet supplemented with Dl-Met supplement at the commercial recommended level for Met; MEM60, MEM70, MEM80, MEM90, and MEM100 treatments: basal diet supplemented with microencapsulated DL-Met at the levels of 60, 70, 80, 90, and 100 % of the commercial recommended levels for Met.

2 Means within a row with different superscripts (a–d) differ (Tukey’s test, *P* < 0.05). SEM indicates the pooled standard error of the mean. *P*-treatment is the overall one-way ANOVA *P*-value comparing all six treatments.

3 *P*-linear and *P*-quadratic are orthogonal polynomial contrasts testing linear and quadratic trends only across MEM60–MEM100 (control excluded), using orthogonal polynomial contrasts.

4 IAA, Indispensable amino acids; DAA, dispensable amino acids.

### 3.5 Antibody response

**Fig. 1:**
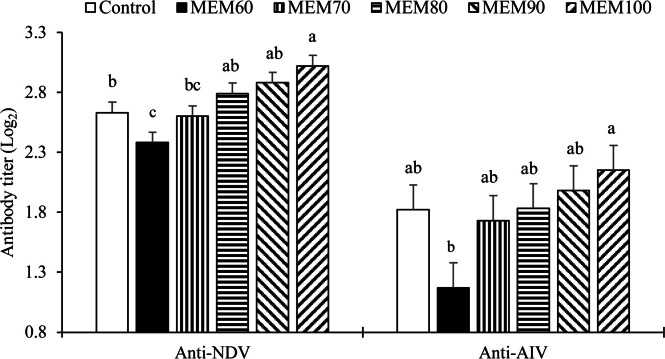
Antibody response (Log_2_) against Newcastle disease virus (**NDV**) and avian influenza virus (**AIV**; H9N2) in broiler chickens fed a basal diet with crystalline DL-Met (control) or microencapsulated DL-Met provided at 60, 70, 80, 90, or 100 % of the commercially recommended Met level (MEM60–MEM100). Bars represent treatment means ± SEM. Different letters above bars denote significant differences among treatments within each gene (one-way ANOVA followed by Tukey post-hoc test, *P* < 0.05).

Based on the antibody titers against NDV and AIV vaccines measured on day 21 (Fig. 1), all experimental treatments increased NDV antibody titers compared with the MEM60 treatment. In addition, the MEM100 treatment resulted in a higher NDV antibody titer compared with the control group. Similarly, the MEM100 treatment enhanced the antibody titer against the AIV vaccine. According to the orthogonal analysis, antibody titers against both vaccines showed a linear increase in response to the incremental levels of the MEM supplement.

### 3.6 Protein deposition-related genes

**Fig. 2:**
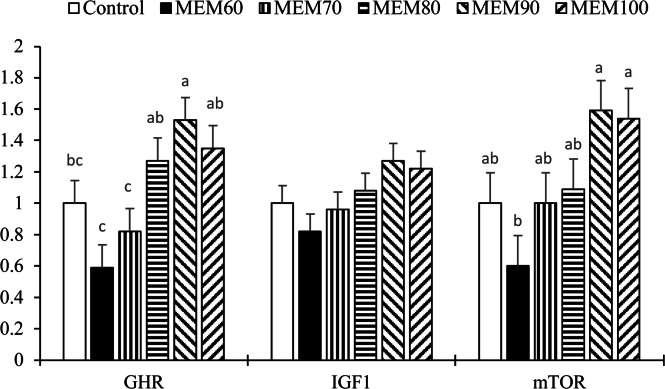
Relative mRNA expression of GHR, IGF1, and mTOR in liver of broiler chickens fed a basal diet with crystalline DL-Met (control) or microencapsulated DL-Met at 60, 70, 80, 90, or 100 % of the commercially recommended Met level (MEM60–MEM100). Bars show treatment means ± SEM (*n* = 7 pens per treatment; RNA extracted from pooled tissue of 3 birds per pen; normalized to **GAPDH**; expression calculated by the 2^−ΔΔCt^ method with the control set to 1.0). Different letters (a–c) above bars indicate significant differences among treatments **within each gene** (one-way ANOVA followed by **Tukey** post-hoc test, *P* < 0.05). Gene abbreviations: **GHR**, growth hormone receptor; **IGF1**, insulin-like growth factor 1; **mTOR**, mechanistic target of rapamycin.

The results related to hepatic expression of growth-related genes (GHR, IGF-1, and mTOR) are presented in Fig. 2. The expression of the GHR gene was significantly higher in the MEM80, MEM90, and MEM100 treatments compared with the MEM70 and MEM60 treatments (*P* < 0.05). A significant increase in the expression of the mTOR gene (*P* < 0.05) was observed, along with a trend towards higher expression of the IGF-1 gene (*P* = 0.077) in the MEM90 and MEM100 treatments compared with the MEM60 treatment.

## 4 Discussion

Met, a key sulfur-containing amino acid, plays a crucial role in protein synthesis, metabolic processes, and immune function, making it essential for the optimal growth of broilers (Khatlab et al., 2019; Zhang et al., 2024). Adequate levels of Met in poultry diets are necessary to support various physiological functions, including enzyme production, antioxidant defense, and tissue repair, all of which affect growth performance in broiler chickens (Pokoo-Aikins et al., 2022; Pang et al., 2023). The growth performance results of this study demonstrate that microencapsulated DL-Met (MEM) supplementation significantly improved BWG and FCR over the entire experimental period (1–42 days). While no significant differences were observed during the early phase (days 1–7), more pronounced effects were evident as the study progressed, with the MEM80 and MEM100 treatments showing higher BWG and better FCR in the later periods. The MEM60 group showed lower performance compared with the control, highlighting that a minimum supplementation of micro-encapsulated DL-Met at around 70–80 % of the control level is required to maintain growth performance comparable with the control group. Interestingly, the MEM90 treatment consistently outperformed the others, exhibiting the highest BWG and the lowest FCR throughout the study, underscoring the effectiveness of micro-encapsulated DL-Met in enhancing growth performance. In contrast, microencapsulated DL-Met supplementation at the 100 % commercially recommended level (MEM100) did not yield superior performance compared with lower inclusion levels, suggesting that exceeding the recommended Met levels does not necessarily provide additional benefits and may result in an imbalance in amino acid absorption. The findings of this study align with previous studies that report improvements in growth performance with encapsulated AA compared with crystalline forms (Liu et al., 2024). It was also found that encapsulating lysine and Met improves their absorption and reduces nitrogen waste in poultry, leading to better growth and feed efficiency (Sun et al., 2020a, b). Moreover, previous studies have demonstrated the advantages of using microencapsulation in different animal models, where encapsulated Met has been shown to improve nutrient digestibility, reduce environmental nitrogen waste, and increase overall feed efficiency (Guo et al., 2020; Liu et al., 2024; Seleem et al., 2024). The benefits of encapsulation in protecting feed additives, like biofactors and antioxidant supplements, have been reported in previous studies because they resist degradation in the stomach, allowing for a slow and controlled release in the small intestine, which enhances their bioavailability and absorption efficiency (Ren et al., 2023; Oretomiloye et al., 2025). This is particularly important for Met supplementation, as encapsulation technology ensures the stability and efficacy of this amino acid, which can lead to improved growth performance in broiler chickens.

This study provides evidence that incorporating microencapsulated DL-Met into the diet at levels ranging from 70 to 100 % of the recommended level effectively maintains the carcass characteristics of the birds. This suggests that the birds did not exhibit any signs of Met deficiency when subjected to these dietary conditions. In contrast, a Met reduction to 60 % of the recommended commercial level, through the utilization of its encapsulated form, resulted in a notable decline in leg yield. Met, an essential amino acid, is known to play a crucial role in a variety of physiological processes, such as protein synthesis, growth, and development (Wilfred et al., 2024). The impact of varying Met levels on carcass characteristics has been the subject of numerous previous studies, which have yielded disparate findings. Multiple studies have reported the negative impact of lower dietary Met levels on broilers' carcass and cut weights (Majdeddin et al., 2019; Rehman et al., 2019). In contrast, Wen et al. (2017b) observed that broilers fed diets with adequate and high levels of Met exhibited higher eviscerated yield and breast muscle yield compared with those fed a low Met diet. It has been suggested that Met supplementation in the diet of fast-growing broilers may enhance their growth through the stimulation of growth hormone secretion and the augmentation of muscle protein accretion (Maynard et al., 2023). Met acts as a methyl‑group donor for creatine synthesis, which is essential for muscle energy metabolism and tissue development (Asiriwardhana et al., 2024). The present study offers additional evidence that supports the reduction in relative abdominal fat observed in broilers fed diets containing 90 % and 100 % micro-encapsulated DL-Met. The observed decrease in fat storage in the MEM90–100 treatments may be attributed to improved Met + Cys balance reducing fat deposition and promoting lean tissue accretion in broilers as reported by de Aguiar et al. (2025).

Based on the findings of the study, manipulating dietary Met levels to 60 % of the recommended intake using the micro-encapsulated form did not result in any significant impact on hematological parameters. To the best of our knowledge, there has been no research conducted to investigate the impact of the encapsulated form of DL-Met on hematological parameters. Supplementation with Met has been shown to modulate hematological parameters, including increases in RBC, WBC, and hematocrit, in broiler chickens fed adequate Met diets (Kachungwa Lugata et al., 2022). In contrast, Zhang et al. (2017) did not find any significant impact of Met supplementation in broiler diets on hematology and the differentiation count of WBC. Furthermore, Tombarkiewicz et al. (2020) proposed that an excessive supply of Met may potentially result in the development of hyperhomocysteinemia, which in turn could lead to a decrease in leukocyte count. The observed variations among studies may be attributed to disparities in environmental conditions, the nature of the Met source used, the dosage levels employed, and the underlying dietary composition.

Postprandial plasma amino acid patterns are a sensitive integrative marker of digestion, intestinal absorption, and splanchnic metabolism in poultry, and they respond rapidly to changes in AA supply and balance (Rochell et al., 2016; Wang et al., 2024). In the present study, replacing conventional DL-Met with microencapsulated DL-Met modified the plasma profile of several AA. Met, Cys, Ser, Ile, and total AA showed a quadratic response, and Lys increased both linearly and quadratically with increasing inclusion of encapsulated DL-Met. The MEM60 diet (60 % of the recommended Met level) produced the highest plasma Met, Cys, and TAA but the poorest growth, indicating that this level of Met was insufficient to support optimal protein deposition and led to accumulation of circulating AA rather than their incorporation into tissue proteins. Similar shifts in postprandial AA balance have been reported when encapsulated Met and Lys were used in hens and broilers, where sustained intestinal release reduced the required inclusion level of crystalline AA without impairing performance (Sun et al., 2020a, 2020b; Liu et al., 2024). These effects are usually attributed to slower luminal dissolution, improved synchrony between AA appearance and energy supply, and potentially altered expression or activity of intestinal AA transporters (Guo et al., 2020; Alabi & Adedokun, 2025).

Despite changes in individual AA, plasma total AA and uric acid concentrations in birds fed the MEM70 diet remained similar to the control, which is consistent with the absence of growth-performance losses at this level of Met reduction. This agrees with recent work showing that carefully balanced low-protein diets supplemented with limiting AA can maintain growth and carcass traits while improving nitrogen-use efficiency (Saleh et al., 2021; Cappelaere et al., 2021). In contrast, broilers offered the MEM60 diet showed both compromised performance and the highest plasma TAA at 42 d, indicating that AA supply or balance had become limiting for protein deposition, leading to accumulation and increased catabolism of circulating AAs. Elevated circulating AA and nitrogenous metabolites such as uric acid have been associated with inefficient AA utilization and excess deamination in broilers (Selle et al., 2021; Alabi & Adedokun, 2025). Taken together, these findings suggest that microencapsulated DL-Met allows a reduction of supplemental Met to around 70 % of current recommendations without impairing AA status or performance, but further reduction to 60 % disrupts the systemic AA profile, decreases the efficiency of AA utilization, and ultimately limits growth.

In this study, broilers supplemented with increasing levels of microencapsulated DL-Met exhibited a linear enhancement in antibody responses against both NDV and AIV (H9N2). This finding aligns with several previous studies that have shown the positive effects of Met on immune function in poultry. Met is an essential amino acid involved in protein synthesis and the regulation of immune responses, particularly through its role in modulating the mTOR pathway, which is crucial for the activation of immune cells and antibody production (Lai et al., 2018; Zarghi & Ghavi, 2024). Furthermore, Met supplementation has been widely documented to improve immune responses by influencing redox balance (Ren et al., 2020; Zhang et al., 2024). Met contributes to the synthesis of glutathione, a major antioxidant that helps mitigate oxidative stress, which is essential for maintaining cellular function and immune responses (Kachungwa Lugata et al., 2022). This antioxidant role of Met may further explain the observed increase in antibody titers, as oxidative stress has been shown to impair immune function in poultry (Ghasemi & Nari, 2023). The observed increase in antibody titers with higher levels of microencapsulated DL-Met in the present study may be attributed to the slow-release properties of microencapsulation technology. This form of Met ensures more efficient absorption and sustained release in the gastrointestinal tract, optimizing its bioavailability and allowing for a prolonged effect on the immune system. In contrast, traditional crystalline Met often leads to a more rapid release, which may not be as effective in maintaining consistent immune activation. The advantages of microencapsulation in improving nutrient utilization and bioavailability have been demonstrated in previous studies (Contreras-López et al., 2024; Oretomiloye et al., 2025), which highlighted the importance of controlled release mechanisms in enhancing immune function in broilers. More conservatively, the linear improvement in NDV and AIV titers with increasing MEM level suggests that adequate Met supply supported humoral immune responses, potentially through Met-dependent pathways related to mTOR signaling (Shan et al., 2024) and antioxidant (glutathione) status (Wen et al., 2017b). A sustained Met supply from MEM may be one contributing factor, but alternative explanations include differences in digestible Met and Met + Cys supply across MEM levels during the starter and finisher phases (Table 1, Table 2).

The regulation of growth through hormonal mechanisms is a complex process that involves intricate interactions among different hormones (Vaccaro et al., 2022). The somatotropic axis, which includes mTOR, GHR, and IGF-I, is widely recognized as the primary regulatory pathway among these hormones (Nawaz et al., 2025). The results of this study demonstrate that microencapsulated DL-Met supplementation significantly modulates the gene expression of key growth-related genes in broilers, including GHR, IGF-I, and mTOR. Met plays a crucial role in regulating protein metabolism (Liu & Kim, 2023), and our findings suggest that encapsulated Met supplementation, especially at the 90 % level, enhances the expression of GHR and IGF-I, which are central to growth regulation in poultry. These results align with previous studies showing that Met supplementation activates mTOR signaling in different animal models (Del Vesco et al., 2015; Irm et al., 2021), which is essential for protein synthesis and muscle growth. The upregulation of mTOR signaling in response to micro-encapsulated DL-Met highlights its role in optimizing nutrient utilization and promoting tissue growth, as mTOR serves as a nutrient sensor that integrates signals from growth factors and AAs to stimulate cell growth and protein synthesis (Shan et al., 2024). Importantly, these molecular responses were consistent with the treatment-level performance outcomes. The MEM treatments that increased hepatic GHR (MEM80–MEM100) and mTOR (MEM90–MEM100) (Fig. 2) corresponded to the treatments showing improved growth efficiency, with the highest 42-d BWG and lowest FCR in MEM90 (Table 4). Biologically, increased hepatic GHR expression may enhance tissue responsiveness to circulating growth hormone (Del Vesco et al., 2015), supporting IGF-1 signaling and downstream anabolic processes, whereas increased mTOR expression is consistent with improved amino acid sensing and activation of translational machinery (Xu & Velleman, 2023), which can contribute to greater protein accretion and improved feed conversion. Furthermore, the observed trend of higher GHR expression in the MEM90 treatment group supports the idea that Met can amplify the effects of growth hormone in broilers, contributing to enhanced growth rates and improved feed conversion efficiency. The interconnected regulation of GHR, IGF-I, and mTOR by Met suggests that microencapsulated DL-Met supplementation acts as a potent growth modulator by influencing multiple pathways involved in muscle development and protein synthesis. Previous studies have shown that Met supplementation can improve the efficiency of protein metabolism by enhancing IGF-I release and activating the mTOR pathway, which promotes muscle growth while inhibiting pathways that lead to protein degradation, such as the myostatin pathway (Wen et al., 2017a; Xu & Velleman, 2023). This integrated response supports the idea that Met is not only a critical building block for protein synthesis but also a signaling molecule that regulates key growth pathways at the genetic level.

Taken together, these results indicate a consistent biological response linking amino acid utilization, immunity, and growth regulation. At the lowest MEM level (MEM60), elevated plasma Met and total AA suggest limited effective Met supply, reduced AA incorporation into tissue protein, and greater circulating AA accumulation. In contrast, higher MEM inclusion—especially MEM90—improved BWG and FCR alongside upregulated hepatic GHR (MEM80–MEM100) and mTOR (MEM90–MEM100), consistent with enhanced nutrient sensing and protein accretion. Although hematological traits were unchanged, NDV and AIV antibody titers increased with MEM level, implying that sustained Met availability supports humoral immune responses. Overall, the parallel improvements in feed efficiency, growth-related gene expression, and vaccine antibody titers provide a plausible mechanism by which microencapsulated Met enhances broiler performance. The significant improvements in growth performance and feed efficiency observed in the MEM90 treatment group further underscore the importance of optimizing Met levels in broiler diets. These findings provide compelling evidence that microencapsulated DL-Met supplementation can enhance poultry production by improving the molecular regulation of growth and muscle development, leading to better overall performance.

## 5 Conclusion

In conclusion, supplying microencapsulated DL-Met at ≥70 % of the recommended commercial level sustained growth performance and carcass yield while allowing an approximate 30 % reduction in supplemental Met. At these inclusion levels, improved performance was accompanied by more favorable plasma amino acid profiles, enhanced antibody responses to H9N2 and Newcastle disease virus, and upregulation of hepatic growth-related genes (GHR, mTOR, and IGF-1), suggesting more efficient utilization of Met and activation of growth-regulating pathways, whereas a 60 % inclusion level was associated with impaired performance and altered amino acid retention. Future studies should further clarify the mechanistic relationships between encapsulated Met delivery, hepatic growth signaling, immune competence, and nutrient partitioning under diverse broiler genotypes and production conditions.

## Ethical statement

The experimental protocol adhered to the guidelines set by the Animal Ethics Committee of Arak University (Approval number 1402-d-13,327) and followed the ARRIVE guidelines 2.0 for animal research, ensuring ethical practices in the treatment and care of the animals. Prior to the initiation of the study, all relevant ethical considerations were thoroughly reviewed, and appropriate steps were taken to minimize animal suffering.

## CRediT authorship contribution statement

**Mohammad Ali Khazab:** Writing – review & editing, Writing – original draft, Visualization, Investigation, Data curation, Conceptualization. **Hossein Ali Ghasemi:** Writing – review & editing, Writing – original draft, Supervision, Software, Methodology, Formal analysis. **Seyed Abdullah Hosseini:** Writing – review & editing, Validation, Project administration, Methodology, Conceptualization. **Iman Hajkhodadadi:** Writing – review & editing, Resources, Methodology, Conceptualization. **Amir Hossein Alizadeh-Ghamsari:** Writing – review & editing, Visualization, Validation, Methodology.

## Declaration of competing interest

The authors declare that the study was carried out without any financial or commercial relationships that could be construed as a potential source of conflict of interest.
